# A Systematic Review of Evidence-Based Family Interventions for Trauma-Affected Refugees

**DOI:** 10.3390/ijerph19159361

**Published:** 2022-07-30

**Authors:** Chansophal Mak, Elizabeth Wieling

**Affiliations:** Department of Human Development and Family Science, College of Family and Consumer Science, University of Georgia, Athens, GA 30602, USA; ewieling@uga.edu

**Keywords:** family, mental health, refugees, traumatic stress, culture, displacement, intervention

## Abstract

Family connections are crucial for trauma-affected refugees from collectivistic cultures. Evidence-based family interventions are consistently promoted to support a host of mental and relational health needs of families exposed to traumatic stressors; however, there is still limited research focused on cultural adaptation and the testing of the effectiveness of these interventions on some of the most disenfranchised populations in the aftermath of forced displacement. This systematic review was conducted to examine the reach of existing evidence-based family interventions implemented with newly resettled refugees globally. Studies included in this review include those testing the effectiveness of a systemic treatment with pre and post intervention evaluation, studies with or without control groups, and studies that include at least one family member in addition to the target participants. Twelve studies met the inclusion criteria. Barriers to conducting randomized control trials with displaced refugee populations are discussed. Recommendations are made for future studies to include a focus on scientifically rigorous multi-method designs, specific cultural adaptation frameworks, and the integration of relational aspects rather than focusing only on individual adjustment. Global displacement continues to rise; therefore, it is imperative that the mental health and wellbeing of displaced populations be treated with a comprehensive, multi-level framework.

## 1. Introduction

Forced displacement estimates exceeded 89.3 million people globally by end of 2021 and is expected to be even higher in the future with new and ongoing global conflicts [1]. The full impact of the COVID-19 pandemic on displacement is yet to be determined. United Nations High Commissioner for Refugees (UNHCR) data showed that arrivals of new refugees and asylum-seekers were sharply down in most regions, which is likely a reflection of how many people were stranded as a result of the pandemic. Forcibly displaced and stateless people are among the most adversely affected groups around the world and continue to face increased food and economic insecurity as well as challenges in accessing health and protection services [1]. Climate change is also driving displacement and increasing the vulnerability of these populations [2]. Many are living in climate “hotspots” where they typically lack the resources to adapt to an increasingly inhospitable environment. The dynamics of poverty, food insecurity, climate change, conflict, and displacement are increasingly interconnected and mutually reinforcing, driving an increasing number of people to search for safety and security [3]. It is also important to note that children account for an estimated 41 per cent of all forcibly displaced people [1].

Forced displacement disrupts the entire community and family structure of migrant and refugee populations [4]. Exposure to multiple traumatic stressors and life adversities are often unavoidable before and during the process of migration [4]. After resettlement in a new country, cumulative daily stressors, additional exposures to traumatic stress over time, poverty, and acculturation stress place refugee families at risk for serious negative mental health outcomes and relational challenges [5,6,7]. The overwhelming consequences of exposure to displacement and traumatic stressors demand multilevel systemic interventions that are culturally responsive while also addressing individual, family relational, and community health needs [8].

Most refugees belong to collectivistic societies that value family connection and interdependence [9]. Family unity and cohesion represent an important indicator of individual mental and relational health in collectivistic cultures [8]. Where there is forced displacement because of human rights violations, organized violence, natural disasters, and climate change in their home countries, refugee families are stripped from their natural contexts and resources and face multiple and enduring losses [8].

The COVID-19 pandemic further exacerbated existing mental and relational health issues; specifically, it created more barriers for refugee families to stay connected when they were geographically separated [10]. Resettlement communities around the world have a sociopolitical and moral responsibility to create infrastructures to support these families. Most importantly, mental health professionals have a critical role in developing and testing interventions to effectively address the mental and relational health of various refugee populations. Despite the overwhelming challenges to survival, these communities have tremendous resilience, and we know historically that when families are provided with opportunities to heal, they recover and thrive in their new countries of resettlement [8].

In 2015, Slobodin and de Jong published a systematic review of family interventions for refugees [4]. This work documented the impact of traumatic stress on individual mental health, the need to interrupt the intergenerational transmission of psychopathology and violence related to trauma exposure, and the need to support family and community healing [4]. Slobodin and de Jong’s study [4] reported that only six experimental studies met their inclusion criteria of family-based interventions, with four being school-based interventions and two being multifamily support groups. They went on to underscore the shortage of research in this area and discussed the challenges of drawing clear conclusions regarding the effectiveness of family interventions for trauma-affected immigrants and refugees. They also called for future trials to go beyond individual-level Post traumatic Stress Disorder (PTSD) treatments and called for a greater focus on family-level processes that incorporate relationships, communication, and resilience. With the increased crisis of global displacement, the focus of this paper is to return to the literature and conduct a follow-up systematic review to further examine studies that test the effectiveness of evidence-based family interventions among trauma-affected refugees globally, as well as to examine cultural adaptation processes and implementation and dissemination strategies utilized across empirical studies. Our purpose was to raise awareness and create a call to action in support of prevention and intervention studies focused on supporting refugee families after resettlement. It is important to evaluate the effectiveness of these family-based programs in terms of scientific rigor and cultural fit for various refugee communities. Specifically, to evaluate the effectiveness of an evidence-based intervention, in addition to measuring clinical outcomes, it is important to examine program implementation and dissemination outcomes to see if there is evidence of promoting or protecting health and preventing ill health in a particular population [11].

### 1.1. Working with Displaced and Minoritized Refugee Families

Displacement can be life threatening for refugee families. Three common stages of migration (i.e., premigration, during, and post migration) are often linked with the development of cumulative traumatic stress among forcibly displaced communities [4,12] often resulting in deleterious mental health and relational maladjustments [13,14]. In premigration, severe traumatic events such as political turmoil leading to mass violence, wars, genocide, human rights violations, as well as natural disasters and climate change have forced people to migrate and seek safety [2,15,16]. During migration, refugees often continue to be exposed to traumatic events through forced displacements both inside and outside their home countries for years [3]. Refugees continue to live in harsh conditions in refugee camps and have to deal with uncertainty and the ambiguity of hope during migration. In post migration, refugees arrive in resettled countries with additional stressors such as family separation, a lack of social support, a lack of employment and language skills, transportation difficulties [17], and limited support from local authorities [14,18]. Additional migration experiences include acculturation stress, severe poverty, living in high crime neighborhoods, and most importantly, living with untreated mental health after exposure to severe adversities before and during their resettlement [19,20,21]. Cumulative traumatic stress at premigration, during, and post migration is associated with psychological and relational consequences such as depression, anxiety disorders, adjustment disorders, PTSD, complicated grief, psychosis, suicide [5,6,7,13,20,22,23,24,25,26,27], the comorbidity of mental health disorders [13,14], the comorbidity of mental health and physical health issues, substance abuse, the disruption of family functioning (e.g., the disruption of couple relationships and parent–child relationships) [6,17,25,28,29,30,31], and the intergenerational transmission of traumatic stress among refugee families [32,33,34].

Mental health professionals working with refugees need to be aware that refugees encounter multiple stressors across all system levels (i.e., individual, family, and community) over prolonged periods of time [8] and suffer from mental health complications due to their comorbid nature [6,13,14,17,25,28,31,35]. At the individual subsystem, exposure to traumatic events during migration leads to extreme stress responses in the brain of affected refugees [36,37,38,39,40]. The amygdala dominates brain functioning and leads to the fragmentation of memory systems as the brain is wired to activate the implicit sensory, physiological, cognitive, and emotional aspects of the traumatic events (associated with the amygdala) without connecting those memories to the context, time, space, and chronology of the events (associated with the hippocampus) that are processed in the prefrontal cortex of the brain [36,37,38]. This fragmentation of memory systems often results in posttraumatic stress symptoms. Individual symptoms of PTSD include re-experiencing, arousal, avoidance, and negative cognitive and affective changes after experiencing life-threatening events or witnessing the life-threatening events of significant others [41]. Trauma-affected individuals tend to isolate themselves, be on guard and hypervigilant, and utilize fear-based coping and avoidance in their daily functioning and relationships [42]. Moreover, trauma survivors may continue to be impaired emotionally, behaviorally, cognitively, biologically, and spiritually long after experiencing the traumatic events [43].

At the family subsystem, exposure to trauma and prolonged family separation during migration disrupts refugees’ family processes [44,45]. Traumatic stress affects not only individuals, but also their families and communities [46,47,48]. The adversities experienced in one system level affects all others as they are interrelated in an ecosystem [8]. These horrifying experiences often impair the individual’s ability to maintain healthy relationships with their family, especially with people who are close to them such as their partners and children [48].

In couple relationships, traumatic stress affects the intimacy and marital satisfaction of trauma-affected individuals. The inability to control one’s emotional and behavioral reactions in response to traumatic memories can lead to anger outbursts [42,49] and all forms of family violence [50,51,52,53]. Specifically, anger outbursts experienced by trauma-affected partners frequently result in intimate partner violence [42,49,54]. This violence is harmful to their relationship as a couple and can be transmitted to subsequent generations as well [55].

In parent–child relationships, trauma-affected parents may employ corporal punishment as a form of child discipline; however, they may not be able to differentiate between punishment as discipline and punishment resulting from their inability to control their anger outbursts [56]. These relational patterns between parent and child are pathways to the intergenerational transmission of traumatic stress among refugee families [32,33,34,44,51,55,57,58]. Having limited to no access to trauma treatment and parenting supports, trauma-affected parents cannot perform their parenting roles adequately [58,59]. As a result, their children are at risk of adverse mental health and relationship consequences such as aggression, low self-esteem, low emotional adjustment, and impulsivity [56,60,61,62], as well as poor school performance, poor peer relationships, violence and delinquency, substance abuse, anxiety, depression, and PTSD [51,52,53,63]. This intergenerational transmission continues to pass on if there are no proper interventions to disrupt its cycle.

Notably, not only do family members, and particularly parents, children, and spouses, influence each other through their adverse experiences; they also influence each other through their strengths and resilience [46,63,64]. Family bonding, a form of family resilience through shared values and interdependence, is a powerful resource for trauma treatment [65]. Moreover, fostering resilience at multi-system levels (i.e., individual, family, and community) is crucial in trauma treatment since resilience in one level affects the other levels too [66,67]. Thus, involving family members in individual and relational trauma treatment is strongly recommended [46,47,48,68].

At the community subsystem level, the resources and support offered by resettlement countries define how fast refugee individuals and their families recover from adversities and cumulative traumatic stress [14]. Local authorities usually fail to provide multi-systemic mental health support to newly resettled refugees [14,18]. Specifically, schools, the main social organizations that work directly with refugee children, often underestimate the complexity of daily stressors that affect their ability to learn and acquire knowledge [69]. At home, witnessing harsh labor conditions, poverty, emotional dysregulation, anger outbursts, and domestic violence between their parents and other family members disrupts the development of refugee children. Child labor is also very common among refugee children and youth, because their labor is often necessary to sustain family functioning. At school, refugee children are prone to being victims of and/or a part of gang violence and delinquency, experiencing discrimination, substance abuse, and a lack of study motivation, and have a lack of educational role models and supports [8,52,69]. All these factors underscore the need for specific systemic interventions to be effectively developed and deployed across all system levels within resettled refugee communities.

### 1.2. Family Interventions Implemented with Trauma-Affected Refugees

A few notable evidence-based family interventions have been adapted for implementation with trauma-affected displaced populations. The interventions have focused on parenting [59,70,71,72,73], multifamily groups [74,75,76,77], and school-based approaches [78,79,80,81,82]. Family-based interventions have proven to be effective in treating traumatic stress and disrupting the intergenerational transmission of traumatic stress among various contexts, but it is difficult to track broad-based effectiveness among refugee populations due to the paucity of family interventions [4]. Most importantly, there is still a lack of culturally adapted or tailored interventions for different ethnic minority refugee populations since most of the evidence-based interventions are based on western and white Euro-American populations [43,47,83].

## 2. Materials and Methods

### 2.1. Search Strategy

We followed the guidelines for systematic reviews suggested by the Preferred Reporting Items for Systematic Review and Meta-Analyses (PRISMA) to identify the studies for inclusion in our systematic review [84]. Our review was registered through PROSPERO as a systematic review with the registration number CRD42022316665. Consistent with the previous systematic review on this topic [4], we also tried to search the Cochrane Central Register of Controlled Trials (CENTRAL), CINAHL, EMBASE, ERIC, Entrez-Pubmed, APA PsycArticles, APA PsycInfo, and the Psychology and Behavioral Sciences Collection. However, because the University of Georgia Library does not have access to EMBASE and Entrez-Pubmed, we replaced EMBASE with the Social Sciences Citation Index and Entrez-Pubmed with PubMed after consulting with the university librarian. As a result, our systematic search sources were APA PsycArticles, APA PsycInfo, the Social Sciences Citation Index, the Psychology and Behavioral Sciences Collection, CINAHL, ERIC, and PubMed. We searched for keywords such as traumatic stress/PTSD, family, prevention/intervention, culture/refugees/immigrants, and displacement/resettlement. Additionally, we incorporated studies included in a previous review article with a similar focus [4]. The search started in June 2013 (when the previous review paused their search) and ended in February 2022.

### 2.2. Criteria for Inclusion and Exclusion

The initial search was conducted by collapsing all of our key terms in each database thus producing a very limited number of articles with the additional limiters of academic peer review, English language, and publication date June 2013 to February 2022. This first search generated 69 articles for screening: four from APA PsycArticles, fifteen from APA PsycInfo, five from the Psychology and Behavioral Science Collection, four from CINAHL, three from PubMed, zero from ERIC, and thirty-eight from the Social Sciences Citation Index. The hand search recommended by the second author who is the expert in the field including the six articles of the previous review by Slobodin and de Jong in 2015 resulted in eleven studies. Table 1 is a brief description of the search procedures.

Because the purpose of this review was to examine the effectiveness of evidence-based family interventions to address traumatic stress among refugee families worldwide, we developed inclusion and exclusion criteria. Our inclusion criteria were: (1) studies that described family-based interventions designed to address individual and relational functioning after trauma exposure and displacement, including pre and post intervention assessments; (2) studies that used Randomized Control Treatment (RCT) designs, non-experimental designs, and feasibility studies that prepared the stage for RCTs; (3) studies that involved more than one family member during the intervention (i.e., not just the target participants); (4) studies that targeted refugees who were affected by traumatic stress (not necessary meeting the PTSD criteria) prior to, during, and after migration; (5) studies that included refugees of all ages; and (6) studies that included interventions that were delivered both in community settings (e.g., refugee camps, schools) and clinical settings (e.g., mental health agencies, hospitals).

The exclusion criteria were: (1) studies that did not target the treatment of traumatic stress symptoms; (2) studies that addressed traumatic stress symptoms among refugee children and youth, but did not involve their caregivers (e.g., parents, grandparents, older siblings, and other family members) during the treatment; and (3) studies that did not examine the effectiveness of a relational intervention, did not address traumatic stress symptoms, or did not intervene at a family level.

## 3. Results

### 3.1. Study Selection

**Figure 1:**
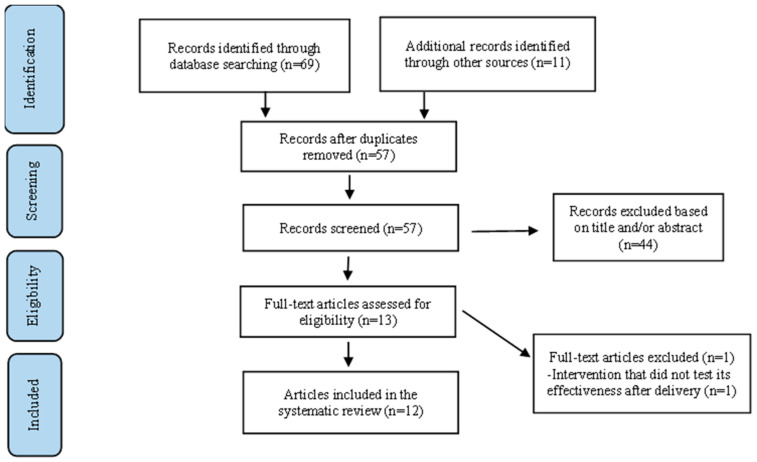
Study Selection Flow Diagram.

The initial search resulted in eighty articles, but only twelve met the full inclusion criteria, including the six articles from the previous systematic review conducted by Slobodin and de Jong (2015). Figure 1 is the summary of our study selection process following PRISMA (2020) guidelines.

### 3.2. Study Designs

Four studies were identified as being in the feasibility testing phase and included assessments and/or qualitative interviews at both baseline and post intervention stages [70,72,74,77]. Five studies were non-experimental and included evaluations at baseline and post intervention without a control group [75,78,79,80]. Three studies were experimental and included assessments at pre and post intervention, and a control group [73,76,81]. One study used a quasi-experimental design by including a control group along with pre and post intervention evaluations [82]. Appendix A demonstrates. Table A1 is a summary description of studies included in this review.

### 3.3. Types of Interventions

#### 3.3.1. Parenting Interventions

Three studies used parenting interventions [70,72,73]. The first study was part of a working group that adapted GenerationPMTO for the context of traumatic stress. The first feasibility study of this adapted parenting intervention was conducted with trauma-affected Acholi mothers in Northern Uganda and demonstrated both acceptability, usability, and limited effectiveness within that population [59]. The working group adapted the intervention for feasibility testing with Karen refugees (from the country of Myanmar) in the U.S. This intervention was grounded in the human ecological model, the social interaction learning theory, and social justice principles [70,71]. GenerationPMTO is an evidence-based parenting intervention adapted in the context of traumatic stress among displaced populations to assist parents in managing their children’s misbehavior. The intervention included nine parenting group sessions for mothers of children ages 5–13. Assessments were conducted with parents and children at pre intervention, post intervention, and at a 3-month follow-up.

The second study used a brief parenting intervention program called Strong Family with the purpose of improving mothers’ parenting skills, mothers’ perceptions of their children, and child behavior [72]. Strong Family is a brief family intervention that consists of three sessions. The total participation time is no more than five hours. In the first week, a one-hour pre group session was conducted among 10–12 caregivers. In the second and third weeks, caregivers and their children first attended separate groups (i.e., a parent’s group and a children’s group) for one hour. Immediately after that, both caregivers and children attended one hour of a family group to conclude the program. The intervention was conducted among 25 Afghanistan refugee families (twenty mothers and five fathers of participating children whose age was between 8–15 years) resettled in Serbia. Assessments were conducted at pre test (t1 at baseline), post test (t2 two weeks after), and follow-up (t3 six weeks after completion) to evaluate the effectiveness of the intervention.

The third study used a parenting and family skills training intervention called Happy Families, adapted from the Strengthening Families Program [73]. The intervention was conducted among 479 Burmese migrant families (i.e., 513 caregivers and 479 children aged 7–15) in 20 communities in Thailand. Happy Families consisted of 12 group sessions. Each session lasted 2.5 h. Caregivers and children attended parallel group sessions followed by joint family sessions. Standardized assessments were conducted at pre and post intervention to evaluate the effectiveness of the intervention through parent–child relationships and family functioning. The follow-up assessments were conducted a month after the intervention for both control and treatment groups, and again six months after the intervention for treatment groups only.

#### 3.3.2. Multifamily Interventions

Four studies employed multifamily group interventions [74,75,76,77]. In 2003 and 2008, Weine and colleagues conducted two studies using multifamily group interventions (i.e., TAFES: Tea and Family Education and Support and CAFES: Coffee and Family Education and Support) that included therapy, psychoeducation, and coping skills for individual and family members in the context of Post Traumatic Stress Disorder among 42 Kosovar refugee families [77] and among 197 Bosnian refugee families resettled in Chicago [76]. Both interventions consisted of nine sessions over 16 weeks. The interventions were grounded in family strength and resilience approaches. The purpose was to assess the effectiveness of the intervention in increasing access to mental health services and decreasing depression. Standardized assessments were conducted prior to and 3 months following the interventions [77], while similar standardized assessments were conducted four times in the 2008 study (at baseline, 6 months, 12 months, and 18 months) [76].

A recent study conducted by Betancourt et al. used a home visiting intervention, the Family Strengthening Intervention for Refugees (FSI-R), that included ten 90-min weekly home-visit sessions among 40 Somali Bantu and 40 Bhutanese refugee families [74]. Similar to the two studies of Wein et al. [76,77], the invention used family strength and resilience approaches grounded in ecological and systemic theories. Standardized assessments were conducted at pre and post intervention to examine the traumatic stress reaction and depression symptoms in children as well as family functioning.

Another recent study conducted by Gotseva-Balgaranova et al. utilized an Evidence-Based Trauma Stabilization (EBTS) that included five psychodrama sessions with children and parent dyads (i.e., 15 children and 16 parents), and four psychoeducation sessions for parents about traumatic stress symptoms and their impact on child development [75]. This study was conducted among seven Iraqi, three Afghan, and five Syrian refugees resettled in Germany and Bulgaria. Psychological assessments were conducted at pre and post intervention to examine the effectiveness of the intervention in reducing PTSD symptoms and depression in both parents and children.

#### 3.3.3. School-Based Interventions

Five studies employed school-based interventions that included caregiver group sessions prior to or/and along with children and youth’s individual and group sessions [78,79,80,81,82]. The first study used the Cultural Adjustment and Trauma Services (CATS) intervention that included relationship building between classroom teachers and CATS staff, outreach services involving cultural brokers as assessors for mental health issues, and clinical services involving psychoeducation, therapy, and family services [78]. CATS is grounded in the Family, Adult, and Child Engagement Services model [79] designed for trauma-affected refugee children and funded by National Child Traumatic Stress Network. The study was conducted among 1049 multiethnic refugee children (only 894 received outreach services, and 149 enrolled in clinical services) from 29 countries resettled in New Jersey in the U.S. Two standardized assessments were conducted at baseline and every 3 months during a three-year period to examine the effectiveness of the intervention in decreasing the symptoms of PTSD and improving functioning.

The second study used International Family, Adult, and Child Enhancement Services (FACES) that included mental health assessment, therapy (individual, group, family), psychiatric services, and support services (e.g., translation/interpretation, travel/transportation) [79]. FACES was initially developed in 1976, and specifically designed for Southeast Asian refugees fleeing Vietnam. The study was conducted with mixed groups of refugee children and youth resettled in the U.S. At the beginning, 97 children and youth participated and only 68 remained at the end of the program, which was conducted over a three-year period. Standardized assessments were conducted longitudinally from December 2003 to August 2005.

The third study used the Trauma Healing Club (THC) intervention adapted from the evidence-based Cognitive Behavioral Interventions for Trauma in Schools (CBITS) [80]. The THC included 12 sessions (i.e., ten CBITS sessions and two drumming sessions in response to the cultural values of African refugees) along with psychoeducation about adverse childhood experiences and their impact on child development for parents and students throughout the intervention implementation. The study was conducted among 88 students and their caregivers who were African refugees resettled in the U.S. Standardized assessments were conducted at pre and post intervention to examine the effectiveness of the intervention in decreasing trauma-related symptoms and increasing coping skills and school performance outcomes.

The fourth study used the Mental Health for Immigrants Program (MHIP) that included eight CBT group sessions for children and youth, two multifamily group sessions for parents along with a child-based intervention, and training about the symptoms and effects of trauma on immigrant children for classroom teachers [81]. The study was conducted among 198 Latinx immigrant children from the third to eighth grade who were diagnosed with trauma-related depression and/or PTSD symptoms. Standardized assessments were conducted at pre and post intervention, and at a 3-month follow-up to evaluate the improvement of PTSD and depressive symptoms.

The fifth study used a multimodal program that included psychoeducation for parents, creative techniques (painting, playing, acting), and relaxation techniques in individual, family and group sessions [82]. The program consisted of twelve sessions over 12 weeks: two information sessions, two diagnostic sessions, six group sessions, two to four individual sessions, and one family session. The study was conducted among 10 Kosovar refugee youth and their parents resettled in Germany. Standardized assessments were conducted at pre and post intervention to examine the effectiveness of the intervention in reducing emotional distress and improving psychosocial functioning among trauma-affected refugee children and adolescents.

We would also like to note an important study that did not meet our inclusion criteria but is directly related to our systematic review. Erdemir conducted a Preschool Education Program (PEP) with the aim of promoting holistic development and boosting school readiness skills before starting primary school among Syrian refugee children resettled in Turkey [85]. The program operated for nine weeks in two schools. The findings showed an improvement in mother–child relationships, positive changes in child behaviors and the mothers’ concepts of their children, as well as positive parenting practices at the end of the program, based on the interviews with the mothers.

### 3.4. Effectiveness of the Interventions/Results of the Studies

Not all interventions in this systematic review measured the same outcomes of PTSD among refugee populations. Six studies reported a reduction in PTSD symptoms (i.e., intrusion, arousal, depression, dissociation, traumatic stress reaction) in children/youth and caregivers at post intervention [74,75,78,80,81,82] in comparison to a control group [80]. Four studies reported the improvement in social functioning among the participants at post intervention [78,79,80,82]. Two studies reported an increase in mental-health-seeking behaviors among participants after completing the program [76,77]. Four studies included family variables (i.e., family hardiness, family problem solving, family comfort in discussing trauma, family arguing, family functioning, and family communication) and they reported positive changes in all family variables [73,74,76,77]. Three studies reported more positive parent–child relationships, especially in parent–child relationship quality, discipline practices (i.e., teaching, directions, emotional regulation, child compliance), and family functioning among participants at post intervention [70,72,73].

### 3.5. Cultural Adaptation Processes

Cultural adaptation is referred to as a process that enhances the fit between the intervention and the target population through the modification, tailoring, and adaptation of intervention elements, while still following the guidelines that contribute to the fidelity and effectiveness of the intervention [86]. There are numerous frameworks to guide the cultural adaptation of an intervention, but these frameworks usually fall into two main categories: (1) the adaptation of intervention content (what to adapt?), and (2) the adaptation process (how/when to adapt, and who should be involved in decision making?). Among the thirteen studies included in this review, only two studies employed cultural adaptation frameworks. The first study, conducted by Ballard et al. [70], used the ecological validity cultural adaptation model developed by Bernal et al. [87]. The cultural adaptation of the evidence-based parenting intervention, GenerationPMTO, also included qualitative needs assessments among stakeholders in the community, intervention development and adaptation focusing on eight ecological dimensions (i.e., language, persons, metaphors, content, concepts, goals, methods, and context), and intervention delivery conducted by trained therapists, intervention coaches, and trained Karen interpreters.

The second study was conducted by Elswick et al. [80], and it used the three steps of cultural adaptation of the intervention (THC) from the original CBITS in response to the needs and cultural values of African refugee families resettled in the U.S. First, African drumming was added into each session to offer emotional regulation during the sessions. Second, the researchers added a pyramid mentoring process developed to foster cultural socialization, cultural identity, and cognitive development through modeling and social support. Third, the original 10-session CBITS was extended to the 12-session THC to ensure the fidelity of the evidence-based intervention despite the additional cultural value of drumming.

Seven studies included in this review did not reference any specific model for the cultural adaptation of the intervention, but focused on different aspects of the relevant culture such as the use of cultural brokers, interpreters, and facilitators who were bilingual and members of immigrant populations [76,77,78,82], observations in their natural setting by program staff to tackle the need for mental health services among refugee children and youth [79], the use of a community-based participatory research approach (CBPR) [77], and the use of initial qualitative interviews to inform the adaptation of the intervention [73].

Three studies did not employ any specific model for the cultural adaptation of the intervention, nor any aspects of the relevant culture. The studies focused on the involvement of parents and teachers in addition to group sessions among students [81], the involvement of parents in four psychoeducation sessions about trauma and five parent–child interaction sessions [75], and the claim that the intervention was already culturally adapted among the population [72].

### 3.6. Implementation and Dissemination Strategies

Implementation science is referred to as methods that help to ensure the accurate translation of research findings and evidence-based practices into community settings with the purpose of improving the quality of health care (i.e., effectiveness, reliability, safety, appropriateness, equity, efficacy) [88]. In other words, implementation science focuses on moving research into practice by incorporating contextual factors and by using multisystemic perspectives. Therefore, implementation and dissemination strategies consider factors that affect the adoption, implementation, and sustainability of a specific intervention in normal settings. According to Fixen et al., the core components of implementations are: (1) staff selection, (2) preservice and in-service training, (3) ongoing coaching and consultation, (4) staff performance assessment, (5) decision-supporting data systems, (6) facilitative administrative supports, and (7) systems interventions [89].

All twelve studies in this review incorporated most of the main components of implementation described by Fixen et al. [89]. Specifically, seven studies conducted preservice training, ongoing consultation and supervision, and regular performance evaluations. For example, the first study, conducted by Ballard et al., included interventionists who were culturally informed intervention coaches and trained therapists committed to working with war-affected and displaced populations [70]. The interventionists adapted the intervention core elements and protocol in response to the needs of the ethnic minority population they worked with by incorporating their cultural values, language, and meaning-making process in the context of displacement. The second study, by Betancourt et al., had a highly trained intervention team consisting of experts in the field (i.e., research assistants, interventionists, licensed clinical social workers, clinical supervisors) and trained staff from two refugee communities [74].

In the third study, by Puffer et al., the intervention team consisted of 40 lay facilitators including staff and non-staff from the implementation organization [73]. The facilitators worked in pairs (one staff and one non-staff), and received an 11-day training. During implementation, staff conducted observation sessions to supervise non-staff. Observers used standardized checklists to evaluate facilitation skills and to determine how much supervision non-staff needed.

In the fourth and fifth studies, led by Weine et al., the intervention team were members of the immigrant populations [76,77]. They received 20 h of implementation training, weekly group and individual supervision, and monthly videotaping of the TAFES and CAFES sessions by an experienced family therapist. In the sixth study, conducted by Birman et al., the intervention team was composed of program assistants who were trained by scale developers, clinicians composed of a doctoral or master level psychologist, an art therapist, a dance therapist, an occupational therapist, a child psychiatrist, and practicum students supervised by licensed staff [79]. Lastly, in the seventh study, by Kataoka et al., the intervention team was composed of school clinicians, educators, and researchers who received 16 h of MHIP intervention training, 2 h of weekly supervision with a psychologist, and 1 h of weekly supervision from an onsite clinical supervisor [80].

Two studies included preservice and in-service training, but did not say much about on-going supervision and performance evaluation during the process of intervention implementation. For example, Beehler et al. developed an intervention team that relied on refugee resettlement staff who were trained in mental health treatments, and CATS clinicians who were bilingual and trained by intervention developers [78]. In the study conducted by El-Khani et al., the intervention team involved two research assistants who were trained by the program developers, and interpreters who were community members [72].

Three studies did not include the delivery of preservice and in-service training among their interventionist team. For example, in the study conducted by Elswick et al., the intervention team was composed of researchers and a clinician, and no other details were provided [80]. In the study conducted by Mohlen et al., the intervention team consisted of a child psychiatrist who led a diagnostic session, a trained medical student who provided therapy, and a Kosovo-Albanian interpreter [82]. Lastly, in the study by Gotseva-Balgarannova et al., the intervention team consisted of trained researchers, EBTS leaders, and an interpreter [75].

## 4. Discussion

This systematic review identified twelve studies that tested the effectiveness of evidence-based family interventions for displaced and trauma-affected refugees. Three studies examined parenting interventions; four studies examined multifamily group interventions; and five studies examined school-based interventions. In terms of study design, only three studies were RCTs and the remaining were non-experimental (i.e., four nonexperimental, one quasi-experimental, and four feasibility studies). No studies compared the effectiveness between individual-based and family-based treatments. Most importantly, only two studies specified the cultural adaptation frameworks they employed while the others simply referred to incorporating exploratory qualitative interviews, cultural brokers, bilingual research teams, and interpreters. The lack of elaboration on cultural adaptation/tailoring and specific dissemination and implementation approaches to target displaced populations points to a shortage of scientifically rigorous and culturally responsive research designs to support individual and relational health among displaced refugees of an ethnic minority.

We would like to frame this discussion as a call to action for those in the mental health field regarding the serious dearth of relational interventions designed and tested to promote healing among refugees following trauma exposure and displacement. It is concerning that despite the knowledge that we face alarming and growing rates of global displacement, we as a prevention and intervention field have not ethically and responsively addressed the mental and relational health of refugee communities. In 2010, the National Institutes of Health assembled a panel to conduct an extensive Delphi study to identify grand challenges in global mental health [90].

The report advanced the following goals: (a) identify root causes, risks, and protective factors; (b) advance the prevention and implementation of early interventions; (c) improve treatments and expand access to care; (d) raise awareness of the global burden; (e) build human resource capacity; and (f) transform health systems and policy responses. Effectively meeting these goals would simultaneously expedite the mental health treatment of refugee communities around the globe. Over a decade has passed since that report was released, yet sustained evidence of growth across those targeted goal areas is missing. We continue to struggle to address the needs of one of the most vulnerable segments of the global population, forcibly displaced refugees.

### Known Barriers to Advancing Mental Health and Systemic Treatments

Design challenges have been pervasive in developing and testing both individual and relational treatments within displaced communities. Gold standards inherent in RCT designs (e.g., control groups, recruitment, blind assignment, statistical power, retention/attrition, dose levels), also create challenges to the effectiveness and superiority of trials and often slow behavioral-based translational sciences. There have been increasing calls to expand our conceptualization of the scientific process to encompass more critical and ethically informed frames that also include deep collaboration with members of the targeted communities [91]. For example, Critical Participatory Action Research (PAR) models specifically incorporate social justice, empowerment, and liberation as part of the scientific endeavor [91,92,93]. The adoption of culturally tailored multi-informant and multi-method research (quantitative, qualitative, and mixed-method approaches) would expand our capacity for developing, implementing, and testing interventions with greater potential for uptake and sustainability within displaced refugee communities [94,95,96,97]. Key researchers in this review also recommended including qualitative studies such as case study methods [79], ethnographic methods [76,77], and community-based participatory research methods [74] along with RCT designs in future research to enhance the effectiveness of family interventions that address mental health and family functioning among diverse displaced refugee families.

Another barrier is the poor resettlement infrastructure in host countries. Considering that the largest percentages of displaced people (86 percent of refugees worldwide) resettled into middle- and low-income countries, limited and often inadequate public and mental health institutions are available to support the resettlement process [3]. Similarly, inadequate infrastructure is also part of the refugee experience in high-income countries [43]. A lack of state policies to systematically assess mental health needs and provide support to resettled families significantly compromises successful family adjustment. For example, a national study conducted by Shannon et al. in the U.S. with 44 refugee health coordinators exploring the mental health training of refugee health coordinators and the systematic screening of refugee mental health reported that they believed it was possible to administer a brief mental health screening during early resettlement meetings; however, only half of the coordinators had received any mental health training [98]. These coordinators identified PTSD and major depression as their top concerns related to refugee mental health and requested training on the mental health needs of arriving refugees. They linked mental health screening with positive referral outcomes for refugee populations. Similarly, a lack of training and awareness of professionals in primary and secondary educational institutions, along with a lack of trained mental health professionals and community health workers, exacerbates concerns and a lack of healing post resettlement. Among the studies reviewed in this paper, Mohlen et al. also highlighted the need to train professionals (i.e., social workers and teachers) who work directly with refugees [82] while Puffer et al. suggested training lay providers who are community members to ensure the sustainability of intervention implementation [73]. Beyond individual assessment and mental health, other studies [59,60,71] documented the broad need for parental support post resettlement as parents feel poorly equipped to navigate new legal, educational, and labor systems.

In addition to the need for greater emphasis on both evidence-based and practice-based interventions for resettled refugee communities (e.g., parenting groups, relational health, peer support), an emphasis on institutional programs that enhance professional capacity, the trauma-focused training of health providers, and community-based refugee centers would go a long way in promoting successful adjustment [99]. Slobodin and de Jong highlighted the need for the implementation of intervention in community settings such as schools, women’s health clinics, or primary care clinics, rather than solely clinical settings, in order to increase the accessibility and cultural responsiveness of mental health services among trauma-affected and displaced refugee families [4].

Most studies in traumatic stress treatment have primarily focused on symptom reduction rather than other aspects of human relationships, such as parent–child relationships, couple relationships, sibling relationships, and both familial and community relationships. Specifically, trauma-affected refugees experience complicated grief and other comorbidities related to mental and relational issues [43], so we advocate for trauma treatments that incorporate multiple systemic factors (i.e., relationship, identity, meaning-making, and community supports) that affect refugee families during resettlement [100]. Several key researchers in this review suggested the inclusion of additional variables in future research in the area of family intervention implementation science: (1) family mental health and functioning along with individual treatment [72]; (2) the cultural components of specific ethnic minority refugees [80]; and (3) timing (e.g., developmental time, family life cycle, time since exposure to trauma, and time of resettlement) [76].

## 5. Conclusions

All studies included in this systematic review report that their programs are somewhat effective in either improving family functioning or reducing PTSD symptoms by comparing the intervention groups and control group or by comparing the pre and post test of the intervention groups. Findings also highlight that culturally adapted, evidence-based family interventions are needed for specific ethnic minority refugee populations to provide multi-systemic support after resettlement. These treatments should also incorporate the specific refugee histories of displacement, traumatic experiences, cultural values, and ethnic identities as part of a broader culturally responsive agenda for resettlement.

## Figures and Tables

**Table 1:** Description of search procedures.

Keywords	Databases	Articles Found
We entered into each database with similar set of keywords: “Traumatic stress” OR “PTSD”, AND “Family”, AND “Prevention” OR “Intervention”, AND “Culture” OR “Refugees” OR “Immigrants”, AND “Displacement” OR “Resettlement”	APA PsycArticles	4
	APA PsycInfo	15
	Social Sciences Citation Index	38
	Psychology and Behavioral Science Collection	5
	CINAHL	4
	ERIC	0
	PubMed	3
	TOTAL	69
	Other sources: Articles that met criteria for inclusion and articles from the previous review by Slobodin and de Jong (2015) [4]	11

## Data Availability

Not applicable.

## Appendix A

**Table A1:** Description of studies included in the review.

Study	Design	Intervention	Aims of the Intervention	Were the Aims Achieved?	Culturally Adaptation Process	Implementation and Dissemination Strategies
Beehler et al. (2012) [78]	Nonexperimental	School-based	Reduce PTSD symptoms Improve social functioning	Yes	Not specified	Refugee staff
2. Birman et al. (2008) [79]	Nonexperimental	School-based	Improve social functioning	Yes	Not specified	Scale developer/therapist/psychiatrist/graduate students
3. Kataoka et al. (2003) [81]	RCT	School-based	Reduce PTSD symptoms	Yes	Not specified	School clinicians/ educators/psychologist
4. Mohlen et al. (2005) [82]	Quasi- experimental	School-based	Reduce PTSD symptoms Improve social functioning	Yes	Use of native translator	Psychiatrist/Medical student/translator
5. Elswick et al. (2021) [80]	Nonexperimental	School-based	Reduce PTSD symptoms Improve social functioning	Yes	Including drumming	Not specified
6. Weine et al. (2008) [76]	RCT	Multifamily group	Increase mental health seeking Improve family functioning	Yes	Use of native facilitators	Refugee staff
7. Wein et al. (2003) [77]	Feasibility	Multifamily group	Increase mental health seeking Improve family functioning	Yes	Use of native facilitators	Refugee staff
8. Betancourt et al. (2020) [74]	Feasibility	Multifamily group	Reduce PTSD symptoms Improve family functioning	Yes	Community participatory research approach (CBPR)	Refugee staff/social workers
9. Gotseva-Balgaranova et al. (2020) [75]	Nonexperimental	Multifamily group	Reduce PTSD symptoms	Yes	Not specified	EBTS leaders/interpreter
10. Ballard et al. (2017) [70]	Feasibility	Parenting	Improve parent–child relationships and family functioning	Yes	Ecological validity model	Trained therapists/Intervention Coaches
11. Puffer et al. (2017) [73]	RCT	Parenting	Improve parent–child relationships and family functioning	Yes	Not specified	Trained lay facilitators
12. El-Khani et al. (2021) [72]	Feasibility	Parenting	Improve child behaviors, family functioning	Yes	Not specified	Trained research assistants/translators
